# Convergent Validation of Methods for the Identification of Psychotherapeutic Phase Transitions in Time Series of Empirical and Model Systems

**DOI:** 10.3389/fpsyg.2020.01970

**Published:** 2020-08-26

**Authors:** Günter Schiepek, Helmut Schöller, Giulio de Felice, Sune Vork Steffensen, Marie Skaalum Bloch, Clemens Fartacek, Wolfgang Aichhorn, Kathrin Viol

**Affiliations:** ^1^Institute of Synergetics and Psychotherapy Research, University Hospital of Psychiatry, Psychotherapy and Psychosomatics, Paracelsus Medical University, Salzburg, Austria; ^2^Department of Psychology, Ludwig Maximilian University of Munich, Munich, Germany; ^3^Department of Dynamic and Clinical Psychology, Sapienza University of Rome, Rome, Italy; ^4^Faculty of Psychology, NCIUL University, London, United Kingdom; ^5^Centre for Human Interactivity, Department of Language and Communication, University of Southern Denmark, Odense, Denmark; ^6^Center for Ecolinguistics, South China Agricultural University, Guangzhou, China; ^7^Outpatient Clinic of Anxiety Disorders and Personality Disorders, Brønderslev Psychiatric Hospital, Brønderslev, Denmark

**Keywords:** self-organization, phase transitions, pattern identification, nonlinear methods, change points, real-time monitoring, phase-transition detection algorithm, PTDA

## Abstract

**Aim:**

In many cases, the dynamics of psychotherapeutic change processes is characterized by sudden and critical transitions. In theoretical terms, these transitions may be “phase transitions” of self-organizing nonlinear systems. Meanwhile, a variety of methods is available to identify phase transitions even in short time series. However, it is still an open question if different methods for timeseries analysis reveal convergent results indicating the moments of critical transitions and related precursors.

**Methods and Procedures:**

Seven concepts which are commonly used in nonlinear time series analysis were investigated in terms of their ability to identify changes in psychological time series: Recurrence Plots, Change Point Analysis, Dynamic Complexity, Permutation Entropy, Time Frequency Distributions, Instantaneous Frequency, and Synchronization Pattern Analysis, i.e., the dynamic inter-correlation of the system’s variables. Phase transitions were simulated by shifting control parameters in the Hénon map dynamics, in a simulation model of psychotherapy processes (one by an external shift of the control parameter and one created by a simulated control parameter shift), and three sets of empirical time series generated by daily self-ratings of patients during the treatment.

**Results:**

The applied methods showed converging results indicating the moments of dynamic transitions within an acceptable tolerance. The convergence of change points was confirmed statistically by a comparison to random surrogates. In the three simulated dynamics with known phase transitions, these could be identified, and in the empirical cases, the methods converged indicating one and the same transition (possibly the phase transitions of the cases). Moreover, changes that did not manifest in a shift of mean or variance could be detected.

**Conclusion:**

Changes can occur in many different ways in the psychotherapeutic process. For instance, there can be very slow and small transitions or very high and sudden ones. The results show the validity and stability of different measures indicating pattern transitions and/or early warning signals of those transitions. This has profound implications for real-time monitoring in psychotherapy, especially in cases where a transition is not obvious to the eye. Reliably identifying points of change is mandatory also for research on precursors, which in turn can help improving treatment.

## Introduction

During the last decades, theories and methods of nonlinear dynamic systems got in the focus of psychotherapy and counseling research. One important quality of nonlinear dynamic systems is their ability to spontaneously create patterns which are not imposed from the outside, but emerge from the interactions of subsystems or parts of a system. The spontaneous emergence of ordered states out of disorder or the transition from one ordered state to another is called *self-organization* (Strunk and Schiepek, 2006; Gelo and Salvatore, 2016). Currently, the most prominent theory and methodology to understand, model, and analyze self-organizing processes is Synergetics (Haken, 2004; Haken and Schiepek, 2010). Phenomenologically, the emergence or transition of patterns takes place in discontinuous jumps, corresponding to “sudden gains” or “sudden losses” in psychotherapy (Stiles et al., 2003; Kelly et al., 2005; Busch et al., 2006; Hayes et al., 2007; Heinzel et al., 2014; Olthof et al., 2019b; de Felice et al., 2019, 2020). Synergetics provides the mathematical framework for modeling and explaining these discontinuous processes (*phase transitions*). In a strict sense, phase transitions occur by shifting one or more control parameter(s) which change the energy dissipation or other conditions of system functioning, e.g., the nonlinear interactions between components or subsystems. Usually, critical instabilities precede transitions, which can be transitions from disorder to order (emergence of one or few order parameters) or from one ordered state to another. In contrast to mathematical modeling (Haken, 2004; modeling of movement transitions: Haken et al., 1985; modeling of psychotherapeutic change dynamics: Schiepek et al., 2017; Schöller et al., 2018) or to physical experiments (e.g., LASER, fluid dynamics, Haken, 2004) in psychological or social real-world systems we often do not know the control parameters and/or cannot manipulate them. Mental processes or emotional functioning are not directly accessible to parameters which can be arbitrarily controlled by an experimenter, a trainer, or a therapist (Schöller et al., 2018). Additionally, control parameters and boundary conditions often are not stable but for their part evolving and unstable, with the consequence that dynamic patterns (attractors) are changing and after a transient period are moving into new patterns. This is what Haken (2004) calls “quasi-attractors.” Given these restrictions of the concept of “phase transitions,” we call changing patterns which do not fulfill all definitory criteria of the concept by the weaker term of “order transitions” (Haken and Schiepek, 2010).

It should be noted that pattern transitions – fulfilling the strict criteria of phase transitions or not – are not only characterized by changes of the mean level of the respective signals, what in psychotherapy research is known as “sudden gains” or “sudden losses,” but by changes of a great variety of dynamic features. This could be transitions from a point attractor to a more or less complex rhythm, from some kind of periodicity to another kind of periodicity characterized by different amplitudes and/or frequencies, from a periodic regime to chaos, or from one type of chaos (e.g., low-dimensional) to another type (e.g., high-dimensional), with transitions in both directions. A systematic classification of transitions is still missing and should be developed in psychotherapy research and other fields of psychology.

In time series characterizing human change processes by psychological measures (e.g., self-ratings), the transition points can be identified by a diversity of methods. Here we focus on methods which can be applied to short time series (100 measurement points or less) and are able to identify pattern transitions and/or precursors of those transitions. Further criteria for the selection of methods is that they should not be restricted by mathematical or parametric assumptions, applicable to real-world time series, and available in computer-based tools for routine process monitoring (e.g., the Synergetic Navigation System, SNS; Schiepek et al., 2018).

The aim of this article is to get an estimate of the validity and stability of different measures indicating pattern transitions and/or early warning signals of those transitions in nonlinear and non-stationary systems. Robust and quantifiable measures of transitions and their precursors are important for science and practice. For science, because research questions focus on the bio-psycho-social multi-level dynamics of phase transitions and the related mechanisms of change (Schiepek et al., 2013) and for practice, because clinical decisions need valid indicators of precursors and early warning signals preceding transitions to the better, e.g., for triggering steps of change, or to the worse, e.g., for preventing suicidal crises. Convergent indicators should help to avoid false positives as well as false negatives in the identification of transitions.

## Materials and Methods

### Methods of Time Series Analysis for the Identification of Transitions

In the following we apply different linear and nonlinear methods of time series analysis to model systems (computer simulations based on mathematical models) and to empirical systems (psychotherapeutic processes assessed by daily self-ratings) undergoing a significant transition.

#### Recurrence Plots (RP)

**FIGURE 1:**
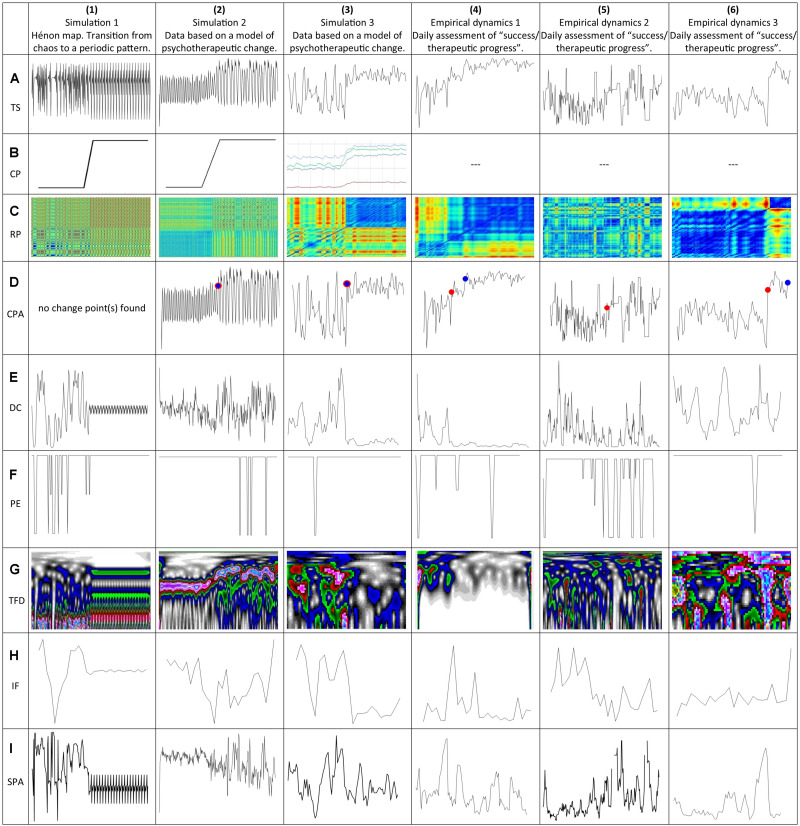
Time series and the applied analysis methods for the detection of critical transitions. All time series were z-transformed to get comparable scales. **(1A)** Transition from a chaotic to a rhythmic regime produced by the Hénon map. **(2A)** Simulation run of a mathematical model of psychotherapeutic change processes with a manually forced parameter shift (time series: “therapeutic progress”). **(3A)** Simulation run of a mathematical model of psychotherapeutic change processes, simulated parameter shifts (time series “insight”). **(4A)** Empirical dynamics of an OCD patient (time series “therapeutic progress”). **(5A)** Empirical dynamics of an MDD patient (time series “therapeutic progress”). **(6A)** Empirical dynamics of an MDD patient (time series “therapeutic progress”). **(1B)** Linear shift of parameter *a* (Hénon map). **(2B)** Linear parameter shifts of all parameters of the model (*a*, *m*, *c*, *r*). **(3B)** Simulated parameter shifts of all parameters of the model (*a*: red, *m*: green, *c*: bright blue, *r*: dark blue). **(1C,2C,3C,4C,5C,6C)** Recurrence Plots (RP) of the time series in line **(A)**. **(1D,2D,3D,4D,5D,6D)** Change Point Analysis (CPA) applied to the time series in line **(A)**; the red dots indicate the identified change points with respect to the mean, blue dots change points with respect to the variance. **(1E,2E,3E,4E,5E,6E)** Dynamic Complexity (DC) applied to all time series in line **(A)**. **(1F,2F,3F,4F,5F,6F)** Permutation Entropy (PE) applied to the time series in line **(A)**. **(1G,2G,3G,4G,5G,6G)** Time Frequency Distribution (TFD) applied to the time series in line **(A)**. **(1H,2H,3H,4H,5H,6H)** Instantaneous Frequency (IF) applied to the time series in line **(A)**. **(1I,2I,3I,4I,5I,6I)** Synchronization Pattern Analysis (SPA) applied to the time series in line **(A)**. CP, (moving) control parameters; CPA, change point analysis; DC, dynamic complexity; IF, instantaneous frequency; PE, permutation entropy; RP, recurrence plots; SPA, synchronization pattern analysis; TS, original time series.

**FIGURE 2:**
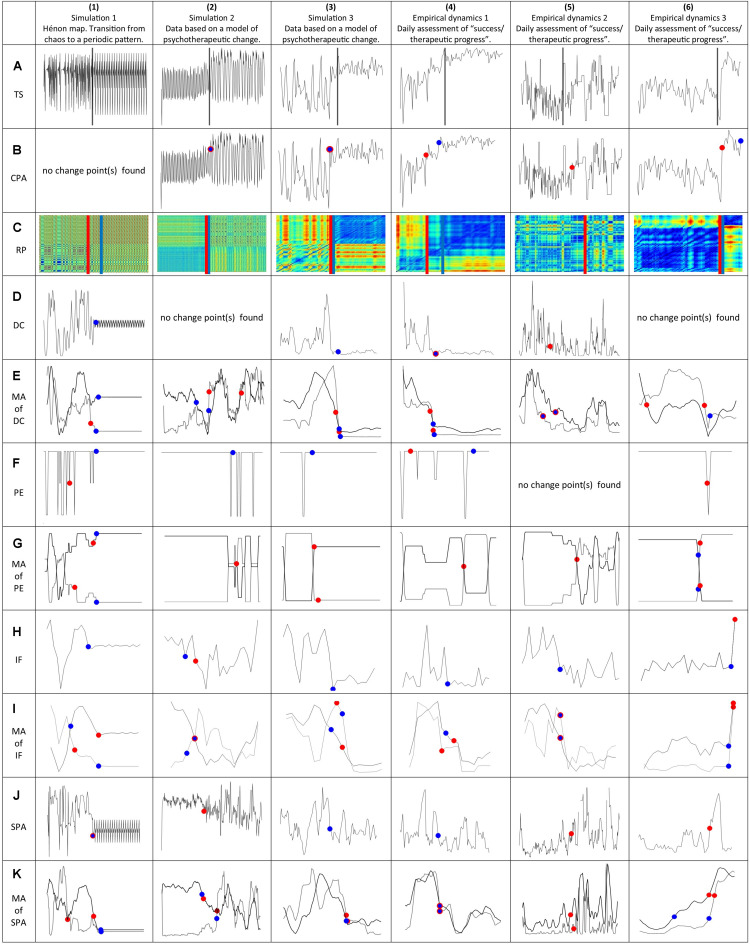
Time series and the applied second order analysis methods for the detection of critical transitions. All time series were *z*-transformed to get comparable scales. The red dots indicate change points with respect to a change of the mean, the blue dots change points with respect to a change of the variance. **(1A,2A,3A,4A,5A,6A)** Original time series, see line **(A)** in Figure 1. The gray bar indicates the mean of all change points. **(1B,2B,3B,4B,5B,6B)** Change Point Analysis (CPA) applied the original time series in line **(A)**. **(1C,2C,3C,4C,5C,6C)** Change Point Analysis (CPA) applied to Recurrence Plots (RP). **(1D,2D,3D,4D,5D,6D)** Change Point Analysis (CPA) applied to Dynamic Complexity (DC). **(1E,2E,3E,4E,5E,6E)** Change Point Analysis (CPA) applied to the moving average (MA, black line) and the moving variance (MV, gray line) of the DC time series (window width: 20 points). **(1F,2F,3F,4F,5F,6F)** Change Point Analysis (CPA) applied to Permutation Entropy (PE). **(1G,2G,3G,4G,5G,6G)** Change Point Analysis (CPA) applied to the moving average (MA, black line) and the moving variance (MV, black line) of the PE time series (window width: 20 points). **(1H,2H,3H,4H,5H,6H)** Change Point Analysis (CPA) applied to Instantaneous Frequency (IF). **(1I,2I,3I,4I,5I,6I)** Change Point Analysis (CPA) applied to the moving average (MA, black line) and the moving variance (MV, gray line) of the IF time series (window width: 5 points). **(1J,2J,3J,4J,5J)** Change Point Analysis (CPA) applied to Synchronization Pattern Analysis (SPA). **(1K,2K,3K,4K,5K,6K)** Change Point Analysis (CPA) applied to the moving average (MA, black line) and the moving variance (MV, gray line) of the Synchronization Pattern Analysis (SPA). CPA, change point analysis; DC, dynamic complexity; IF, instantaneous frequency; MA, moving average; MV, moving variance; PE, permutation entropy; RP, recurrence plots; SPA, synchronization pattern analysis; TS, original time series.

This method identifies recurrent patterns of time series in a time × time diagram (Eckmann et al., 1987; Webber and Zbilut, 1994). A recurrence plot is a square matrix that visualizes times at which a pattern of a dynamical system is identical or very similar to a pattern that has occurred before. In time series of one variable, as in our examples, the pattern of the system is identical to several consecutive values of the variable. For example, one pattern could be “linear increase,” another one “increase and decline.” The color of each element of the matrix indicates the similarity between the patterns at each time: in the recurrence plots of Figures 1, 2 blue indicates similar (recurrent) patterns, red very different patterns. The color of the matrix thus indicates times where the pattern of the time series changes.

Technically, snippets of a full time series are embedded in a phase space with time-delay coordinates. This implicates that the number of time-delay embedding coordinates, corresponding to the snippet length, and the time delay τ between the embedded measurement points have to be defined. Usually, τ is defined by the first zero-crossing or the first minimum of the autocorrelation or of the transinformation function of the time series. By this method, each snippet of the time series is embedded in the time-delay phase space by a vector point. The cell entries in the time × time Recurrence Plot can be the Euclidean distances between the vector points (distance matrix) which are rainbow color-coded with blue = recurrent to red = transient, or, the distances can be binary coded according to a selected threshold. Technically, this threshold corresponds to the radius of a hypersphere^1^ which defines which other vector points are “neighbors,” that is, similar or “recurrent” segments of the dynamics. In a Recurrence Plot, recurrent patterns and their transients become apparent. For all Recurrence Plots we defined 3 time-delay embedding coordinates for each time series with τ = 1.

#### Change Point Analysis (CPA)

The method (Killick et al., 2012) is sensitive to changes of specific statistical properties of a time series. A time series *x* contains a change point if it can be split into two segments *x*_1_ and *x*_2_ such that *C*(*x*_1_) + *C*(*x*_2_) + *k* < *C*(*x*), where *C* represents the cost function, here *C*(*x*) = *Nvar*(*x*), *k* is a threshold, and *N* the number of time points of *x*. In mathematical optimization, a cost function determines how well the data fit a certain assumption. A popular example of a cost function is the Mean Sum of Squares; here, the cost function is simply the variance of a section of the time series [*var*(*x*)] times the number of points constituting this section (*N*). In other words, a change point is detected between the segments *x*_1_ and *x*_2_ of a time series, if the sum of the variance of the statistical property of interest, e.g., the mean of the segments, is smaller than the variance of this property of the whole time series; otherwise, no change point is detected. In our application, a maximum of two change points was allowed, one for detecting changes of the mean and one for detecting changes of the variance. The analysis was done with the function *ischange* implemented in Matlab (Release 2018b) with the default threshold.

Consider the time series {2,2,2,4,4,4,4,4,4,4}, where the mean changes from 2 to 4 between *t* = 3 and *t* = 4. The change point analysis algorithm first splits the time series into two segments, *x*_1_ from *t* = 1 to *t* = 2, and *x*_2_ from *t* = 3 to *t* = 10. For both segments, the cost function *C* is calculated: the first part includes *N* = 2 time points, the second part *N* = 8 time points with *var*(*x*_1_) = *0* and *var*(*x*_2_) = *0.5*, hence *C*(*x*_1_) = 2⋅0 = 0 and *C*(*x*_2_) = 8⋅0.5 = 4. The sum of *C*(*x*_1_) and *C*(*x*_2_), 4, is then compared to the cost function of the whole time series, *C*(*x*) = 10⋅0.933 = 9.33. Since *C*(*x*_1_) + *C*(*x*_2_) + *k* are not less than *C*(*x*), the algorithm concludes there is no change point when segmenting the time series after *t* = 2. It then proceeds by splitting the time series between *t* = 3 and *t* = 4 and repeats the tests for these segments. Now, both the variance of *x*_1_ and *x*_2_ are zero, hence *C*(*x*_1_) + *C*(*x*_2_) + *k* < *C*(*x*) – a change point is detected correctly between *t* = 3 and *t* = 4.

#### Dynamic Complexity (DC)

Dynamic Complexity (DC) (Schiepek, 2003; Haken and Schiepek, 2010; Schiepek and Strunk, 2010) was developed to identify critical instabilities in short and coarse-grained real-world time series, without further mathematical or parametric assumptions. DC mirrors the increased complexity and sensitivity to noise and perturbations of system dynamics before phase transitions, but also the fact that regimes or attractors of human dynamics realize different degrees of complexity (e.g., emotional rigidity in Major Depressive Disorder or emotional instability in Borderline Personality Disorder). DC is the multiplicative product of a fluctuation measure and a distribution measure applied to discrete time series data with given data ranges [*x*_*min*_, *x*_*max*_] and constant discrete time intervals between the data points (sampling frequency, e.g., one observation per day). The fluctuation measure (*F*) is sensitive to the amplitudes and frequencies of a time signal, and the distribution measure (*D*) scans the scattering of values or system states occurring within the range of possible values or system states. In order to identify non-stationarity, DC is calculated within a data window moving over the time series. Because the empirical time series we use in this methods test were collected by daily ratings, we apply a window width of 7 measurement points.

#### Permutation Entropy (PE)

Like Dynamic Complexity, this measure (introduced by Bandt and Pompe, 2002) identifies complexity in natural, real-world time series without restricting parametric assumptions and with high tolerance for noise. Applied to the dynamics of some chaotic model systems like the logistic map, PE behaves like the positive Lyapunov exponent (Bandt and Pompe, 2002; Schiepek and Strunk, 2010). PE is calculated by studying the frequency distribution of value sequences within a moving window. Patterns are constructed from the data in the moving window on the basis of so-called “words” with a given word length *n*. All possible words of length *n* within the moving window are investigated for their rank ordered sequences, and values within a word are recoded in rank order numbers ranging from 0 to *n* − 1. Therefore, a permutation of *n*! rank number patterns can theoretically be found within a data set with no ties. Permutation Entropy depends on the window width and the word length *n*. Permutations of word length 3 are calculated for a window width of 7. The calculations were done with the permutation entropy toolbox for Matlab (Ouyang, 2019).

#### Time Frequency Distribution (TFD)

Time frequency distribution (TFD) is a method to calculate and visualize the frequency of a signal (time series) as it changes with time (Cohen, 1989; Sejdić et al., 2009). In order to identify frequency changes, a moving window approach is implemented. Mathematically, both time *t* and frequency ω are variables of a distribution *P*(*t*,ω) which describes the amplitude (energy) of the signal at each given *t* and ω. Here, we use the so-called Stockwell transform (*S*-transform) which is a combination of two common TFD-methods, the Short Time Fourier Transform and the Continuous Wavelet Transform (Stockwell et al., 1996). It preserves the phase information available from the former method but uses the variable (i.e., not fixed) window length of the continuous wavelet method. For visualization, time and frequency are plotted on a plane (*x*: time, *y*: frequency) and color-coding is used for the representation of the amplitude (energy) of the frequencies.

#### Instantaneous Frequency (IF)

The IF of a non-stationary signal is a time-varying parameter that relates to the average of the frequencies present in the signal as it evolves (Boashash, 1992a, b). It reduces the TFD matrix to one dimension by estimating the first conditional spectral moment of the TFD, which represents the average of the frequencies at each time point. IF was calculated using the function *instfreq* implemented in Matlab (Release 2018b).

#### Synchronization Pattern Analysis (SPA)

An increase of the synchronization of subsystems or components of a system before critical transitions was observed in ecosystems (Dakos et al., 2012), in the emergence of diseases (Chen et al., 2016), and in psychotherapeutic processes (Haken and Schiepek, 2010; Schiepek et al., 2016b). Here we use the absolute (sign-independent) values of the Pearson correlations between the variables of the systems under investigation. For the model systems, the variables were the one shown in Figures 1 and 2 as well as the other variables of the system; for the empirical time series, the variables were the items constituting the factor shown in Figures 1 and 2. The absolute values of the [*N*(*N* − 1)/2] correlations (*N* is the number of variables) are averaged within a moving window (window width = 7). This time varying averaged correlation is a measure of the coherence of the system dynamics.

### Quantification of Transitions

The application of these different methods allows to detect aspects of the original time series that go beyond a shift of the mean and/or variance, i.e., changes in the frequency (TFD and IF), recurring patterns (RP), entropy (PE), critical fluctuations (DC), and synchronization (SPA) (Figure 1). In order to quantify changes in these aspects, Change Point Analysis is applied to the results of these first step analyses (Figure 2). In addition, the Change Point Analysis was applied to smoothed versions of the first step analyses that yielded one-dimensional time series (i.e., the Dynamic Complexity, Permutation Entropy, and Instantaneous Frequency). For the Instantaneous Frequency, a gliding window with 5 points was used, since the methods results in less data points than the original time series, and gliding windows with 20 points for the other methods. These time series of the moving average (MA) and the moving variance (MV) were again analyzed with the Change Point Analysis. As a first approximation to a quantification of the Recurrence Plots, we applied the Change Point Analysis to each line of a Recurrence Plot and used the arithmetic mean of the resulting change points as the change point for the whole Recurrence Plot. The localization and concentration of all second order change points is listed in Table 1.

**TABLE 1:** Localization and analysis of the change points.

	**Hénon (1a)**	**Simulation 1 (2a)**	**Simulation 2 (3a)**	**Empirical case 1 (4a)**	**Empirical Case 2 (5a)**	**Empirical Case 3 (6a)**
Length of time series	300	300	101	111	282	80
Real phase transition	125–150	100–150	45–58	Unknown	Unknown	Unknown
Change point analysis applied to…						
Original time series	–	145 twice	50 twice	31 and 46	146	65 and 79
DC	152	–	57	38 twice	86	–
MA of DC	147	113 and 240	66 and 69	47 and 50	102 twice	25
MV of DC	151 and 167	148 and 149	69 and 70	50 and 51	69 twice	56 and 59
PE	76 and 153	208	32	13 and 86	–	61
MA of PE	157 and 166	–	45	–	–	54 and 55
MV of PE	105 and 166	226	48	76	165	54 and 55
IF	139	80 and 110	56	60	118	76 and 80
MA of IF	74 and 164	85 twice	53 and 61	53 and 65	114 twice	76 and 80
MV of IF	84 and 164	65	57 and 61	45	123 twice	76 and 80
SPA	145	125	49	45	144	56
MA of SPA	152 and 172	132 and 136	68	51 twice	142	59 and 40
MV of SPA	79 and 173	173 and 174	68 and 69	51 twice	151	59 and 62
RP	135 and 172	129 and 133	51 and 52	33 and 45	173	64 twice
Mean (*SD*)	142 (34)	140 (48)	57 (10)	49 (15)	121 (31)	62 (14)

*The change point analysis was applied to the “second step” measures presented in Figure 2. When two numbers are given, one refers to a change point found with respect to a change of the mean, the other found with respect to a change of the variance. When no change point was found, this is indicated by ‘–’. The means (last line) correspond to the vertical gray bars in Figure 2(A). DC, dynamic complexity; IF, instantaneous frequency; MA, moving average; MV, moving variance; PE, permutation entropy; RP, recurrence plot; SD, standard deviation; SPA, synchronization pattern analysis.*

### Time Series of Model and Empirical Systems Used for the Identification of Phase Transitions

In order to identify phase transitions, we prepared six time series from model systems and from empirically assessed psychotherapy processes. The model systems are used to create artificial or simulated phase transitions which fulfill the definition criteria of a phase transition created by at least one moving control parameter. The empirical time series reveal pattern transitions without knowing the responsible control parameters.

The first dynamics [Figure 1(1A)] was realized by the Hénon system, which is – like the logistic map – a well know two-dimensional nonlinear map creating oscillatory and chaotic patterns: $x_{k+1}=y_{k}+1-a\,x_{k}^{2}$, *y*_*k* + 1_ = *bx*_*k*_. 124 iterations were produced with initial values *x*_0_ = 0, *y*_0_ = 0 and parameter values *a* = 1.20, *b* = 0.30. From iteration 125 to 150 the value of the parameter *a* was linearly shifted to *a* = 1.25 (step width: 0.002), *b* was left unchanged.

After this shift, further 150 iterations were produced at constant parameter values. Depending on the shift of parameter *a*, the dynamics moved from deterministic chaos to a regular rhythm. Figure 1(1A) shows the dynamics of variable *x*.

The second example of a phase transition [Figure 1(2A)] was created by a mathematical model of psychotherapeutic change processes (Schiepek et al., 2017). The model includes five variables (S: success and therapeutic progress, M: motivation for change, I: insight and getting new perspectives, E: emotions, represented by a bi-dimensional scale between dysphoric and positive emotions, P: problem intensity and symptom severity), which are interconnected by 16 nonlinear functions constituting the terms of five coupled nonlinear equations (one for each variable). Four control parameters, which can be understood as competencies or dispositions of a patient mediate the interactions between the variables. Depending on their values, the effect of one variable on another is intensified or reduced, activated or inhibited. The parameters are *a:* working alliance and capability to enter a trustful cooperation with the therapist, *c:* cognitive competencies, capacities for mentalization and emotion regulation, *r:* behavioral resources and skills for problem solving, *m:* dispositional motivation for change, self-efficacy, and reward expectation^2^. For creating the chaos-to-chaos phase transition, 100 iterations were simulated with initial values of *S* = −40.7, *M* = 7.5, *I* = 100, *E* = 97.6, *P* = 61.5, and parameters *a* = *c* = *r* = *m* = 0.20, then all parameters were moved by a linear shift from 0.20 to 0.35 between iterations 100 and 150, and after this (from iteration 151 to 300) the parameter values were kept constant. Figure 1(2A) represents the variable S (“success and therapeutic progress”), normalized by a *z*-transformation. During the simulation 5% dynamic white noise was added on all variables.

The third example of a phase transition [Figure 1(3A)] was produced by the same model system as the second. The difference is that the parameters were not forced to move by a manipulation, but by a simulation of the parameter dynamics. For this extended simulation model four nonlinear equations for each of the four control parameters were added, which were coupled to the five order parameter equations. At a specific period (between iteration 44 and 57) the control parameters underwent a sudden increase with a steeper gradient as before and after. The initial conditions of the variables were: *E* = 97.6, *P* = 61.5, *M* = 7.5, *I* = 100, *S* = −40.7, the initial values of the control parameters were *a* = 0.10; *c* = 0,75; *r* = 0,46; *m* = 0.53. The dynamics was driven by a continuous dynamic noise input of 15% on *E* and *P*, and of 10% on *M*, *I*, and *S*. The phase transition results from a circular causality between the coupled dynamics of the variables (order parameters) and the control parameters. If this circularity crosses a self-organized threshold a phase transition takes place (Schöller et al., 2018). It occurred between iteration 44 and 57, where the parameters realized a steep increase from *a* = 0.11, *c* = 0.72, *r* = 0.48, *m* = 0.55 to *a* = 0.22, *c* = 0.96, *r* = 0.77, *m* = 0.89. In Figure 1(3A) the variable *I* (“insight/new perspectives”) is shown.

The examples four, five, and six are taken from a real-time monitoring of psychotherapeutic change. The male patient (diagnosis: Obsessive-Compulsive Disorder, OCD) of example four was treated in an inpatient setting (combining Cognitive Behavior Therapy with different group and creative therapies) and underwent a sudden gain in OCD- and depressive symptoms after about the first third of the therapy. The assessment was realized by daily self-ratings of the items of the Therapy Process Questionnaire (TPQ, Schiepek et al., 2017) presented by the internet-based Synergetic Navigation System (SNS, Schiepek et al., 2018). The TPQ included 47 items distributed on 7 subscales. In Figure 1(4A) the time series of the factor S “success/therapeutic progress” is shown, which corresponds to the factor S of the mathematical simulation model [Figure 1(2A)].

The female patient (diagnosis: generalized anxiety disorder, together with different comorbid diagnosis such as somatization, depression, PTSD, OCD, personality disorder with dependent, borderline and histrionic traits) of example five was treated in an outpatient setting (weekly single therapy sessions and a parallel group program, both with focus on Mentalization-Based Therapy adjusted to anxiety disorders) and underwent a transition in her development after about the first half of the therapy. The assessment was also realized by daily self-ratings using a Danish translation of the TPQ, presented by the SNS. In Figure 1(5A) the time series of the factor S (“success/therapeutic progress”) is shown, which corresponds to the variable S of the mathematical simulation model [Figure 1(2A)].

The male patient (diagnosis: Major Depressive Disorder) of example six was – like the patient of example four – treated in an inpatient setting (combining Cognitive Behavior Therapy with different group therapies, especially psychodrama, mentalization-focused therapy, skills training, and creative therapies). He underwent a transition in his development at the very end of the hospital stay. The phase transition was not preceded by a critical instability, but had the shape of a transient relapse, i.e., a short period of deterioration followed by a sudden gain. The assessment also was realized by daily self-ratings using the TPQ. The case and the synergies of different therapeutic experiences preparing the phase transition were described in detail in a single case study (Schiepek et al., 2018). In Figure 1(6A) the dynamics of the factor *S* (“success/therapeutic progress”) is shown, which corresponds to the factor *S* of the mathematical simulation model [Figure 1(2A)].

### Statistical Analysis

Two methods were used to investigate if the change points were clustered within a certain range of the time series instead of being randomly distributed. Inspired by bootstrapping, random values were drawn from an equal distribution of the length of the respective time series with the *unidrnd* function implemented in Matlab (version R2018b). The number of random values drawn each time was equivalent to the number of change points found in the second order analysis for the respective time series, e.g., for the Hénon map, 23 change points were drawn randomly 100 times from a uniform distribution. These sets of change points are randomly distributed onto the time series and have nothing to do with the phase transition of the time series. When comparing the dispersion of the random change points with the dispersion of the real change points, it is possible to statistically assess if the real change points accumulate, i.e., their dispersion can be tested against randomly spread points.

In a first approach, the interquartile range (IQR) was calculated for the real change points and the 100 sets of random change points for each time series. The IQR describes the number of points within the second and third quartile of the data, i.e., the inner 50% around the median, and is a measure of the dispersion of the data comparable to the variance of normally distributed data. Then, the confidence intervals of the IQRs of the random data were calculated in order to see if the IQR of the real data lies within the confidence intervals.

In the second approach, the dispersion of the change points was assessed by fitting a normal distribution to the change points, using the *fitdist* function with option “Normal” in Matlab. This was done separately for the 100 sets of random change point samples and the real sample for each time series. The width of the normal distribution is characterized by the standard deviation σ. The estimated standard deviation of each fitted normal distribution was used as a measure of dispersion. For the sets of random change points, the mean and the 95% confidence intervals were calculated. If the dispersion of the real change points was significantly lower than those of the random change points, their standard deviation σ would not lie within the confidence interval of σ of the random change points. This would indicate that the real change points cluster around a certain section of the time series, in contrast to random points that have nothing to do with the phase transition period of the respective time series.

## Results

### Identified Phase Transitions

**FIGURE 3:**
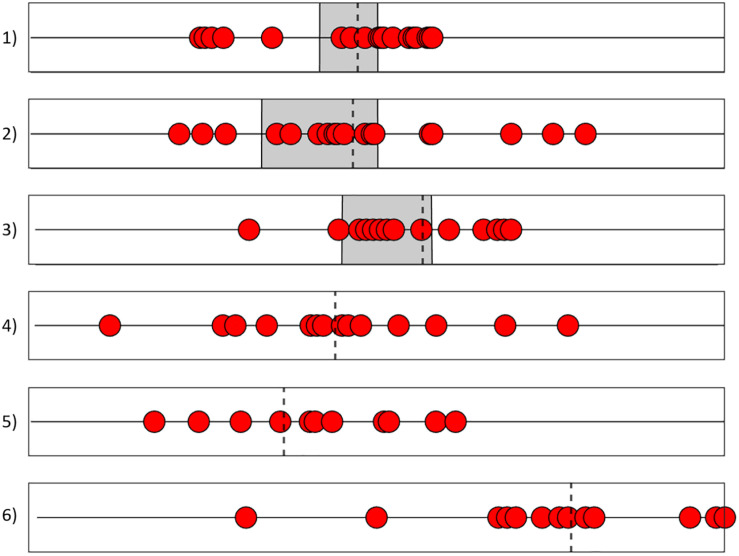
Summary of the change points found by the different analysis methods for all 6 time series (Figures 1, 2). The red dots mark the change points from Table 1. The dashed line marks the mean of all change points, and the gray square indicates the known phase transitions in the simulated time series.

For each time series, phase transitions were assessed by means of change point analysis applied to several nonlinear analysis methods (Figures 1, 2). A summary of the exact values of the change points is given in Table 1, and a visualization in Figure 3. Note that the change point analysis alone is not able to identify all changes, e.g., this method did not find a change in the original time series of the Hénon map (1A). The CPA algorithm is designed to detect changes of the variance or of the mean of the time series; it is not able to detect changes of rhythms or patterns in general. For the simulated data (1A/2A/3A), where the points of the phase transitions are known, most analysis methods yielded points within the real phase transitions and were thus able to successfully identify the real points of change. For the empirical time series (4A/5A/6A), the different methods also suggested points that are consistent with the visually visible transitions. The mean of all change points found with the different methods per time series (last line in Table 1) lies well within the real phase transition. The combined application of all methods is able to reliably identify the real phase transitions.

### Comparison With Random Data

As described in the Section “Materials and Methods,” two approaches were applied to test if the change points found in the empirical data are clustered around a certain region of the time series, or if they were randomly distributed. The first method calculated the interquartile ranges (IQR), which describe the dispersion of the change points onto the time series. The confidence interval for the IQRs of the 100 randomly distributed values, and the IQR of the empirical data is given in Table 2. For all time series, the IQR values of the original time series are much smaller and lie well outside the confidence intervals of the IQRs of the random data. It can be concluded that the change points of Table 1 are not randomly distributed but cluster around the real phase transitions of the respective time series.

**TABLE 2:** Analysis of the interquartile intervals (IQR) of the empirical and the random data, expressed as % of the length of each time series.

	**Hénon map (1a)**	**Simulation 1 (2a)**	**Simulation 2 (3a)**	**Emp. case 1 (4a)**	**Emp. case 2 (5a)**	**Emp. case 3 (6a)**
Mean IQR of random data	47%	47%	47%	48%	48%	48%
CI of IQR of random data	[45%, 49%]	[45%, 49%]	[45%, 49%]	[46%, 50%]	[46%, 50%]	[46%, 50%]
IQR of original data	10%	17%	17%	5%	19%	26%

*The empirical IQRs are considerably smaller than the random IQRs and lie well outside the 95% confidence interval of the random IQRs. In other words, the random data cover around 45–50% of the length of the respective time series, while the change points of the original data cover only 5% (in the best case) to only 26% (in the worst case), i.e., they are much more concentrated on a certain section of the time series. This indicates a non-random distribution of the change points found by the algorithm. CI, confidence interval; IQR, interquartile range.*

**FIGURE 4:**
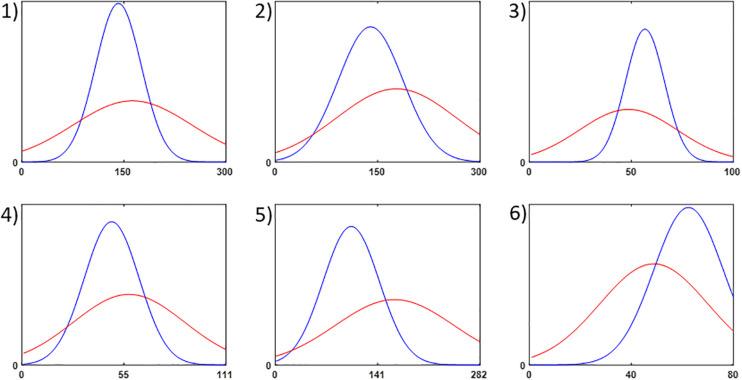
Illustration of the second statistical analysis method: normal distributions were fitted to all sets of change points. The blue line represents the distribution for the real change points found by all methods, and the red line those of the random samples. The width of the fitted distributions allows to conclude that the real change points are much more concentrated (clustered) on a certain section of the time series, since their distributions are considerably narrower.

This result is confirmed by the second analysis method, which fits a normal distribution to the change points (Figure 4). Table 3 gives the mean standard deviations (σ) of the random CP sample and the σ of the real. As anticipated, the mean σ of the random sample is larger than the σ of the real sample for each time series. The large values of the σ were expected since a normal distribution was fitted to equally distributed data. The statistical difference of the σ was assessed by the 95% confidence intervals of the random data. Table 3 shows that the real σ lie outside the confidence intervals of the random data, i.e., they are significantly different. In other words, the σ of the random change points differed significantly from the σ of the empirical data for all time series.

**TABLE 3:** Mean and 95% confidence intervals of the width of the normal distributions (σ) fitted to the random change point samples and the original sample, expressed as % of the length of each time series.

	**Hénon map (1a)**	**Simulation 1 (2a)**	**Simulation 2 (3a)**	**Emp. case 1 (4a)**	**Emp. case 2 (5a)**	**Emp. case 3 (6a)**
Mean σ of random samples	29%	29%	24%	27%	29%	26%
CI of σ of the random samples	[22%, 40%]	[22%, 42%]	[22%,40%]	[22%, 41%]	[22%, 43%]	[22%, 40%]
σ of the original sample	11%	16%	10%	13%	14%	17%

*The empirical σ are considerably smaller than the random σ and lie well outside of their 95% confidence intervals. In other words, the random data cover around 22–43% of the length of the respective time series, while the change points of the original data cover only 10% (in the best case) to only 17% (in the worst case), i.e., they are much more concentrated on a certain section of the time series. This indicates a non-random distribution of the change points found by the algorithm. CI, confidence interval; σ, standard deviation (width of the fitted normal distribution).*

To exclude effects of a possible oversampling, the tests were repeated without the change points found by the CPA on the gliding mean and gliding variance methods (lines E, G, I, and K in Figure 2). The results remained the same, i.e., the IQRs and σ of the real data were well outside the 95% confidence intervals of the random data.

## Discussion

A diversity of methods which were expected to be able to identify critical transitions in time series were applied to simulated (Hénon map and two kinds of simulation runs of a mathematical model of psychotherapeutic change) and naturalistic processes (daily self-assessments of three patients during a psychotherapeutic process). The three examples of phase transitions in simulated data were used because in these cases the periods of the transitions are known and can be objectively localized. The following methods were used for time series analysis: Recurrence Plots (RP), Dynamic Complexity (DC), Change Point Analysis (CPA), Permutation Entropy (PE), Time Frequency Distribution (TFD), Instantaneous Frequency (IF), and Synchronization Pattern Analysis (SPA). In a further step we applied CPA to the time series of DC, PE, IF, and SPA, as well as to the moving average and the moving variance of the DC, PE, and IF dynamics. The results show that the methods are convergent in terms of the identification of the critical transitions of the process. The arithmetic average of the change points of these second order dynamics is placed within the windows of shifting control parameters [examples 1, 2, and 3, see Figures 1(1A,2A,3A), 3], or in close neighborhood to these known shifts. In the empirical dynamics, the change points are placed in a very narrow range compared to the time series length. The convergence of all methods is obvious and highly significant for all time series (Tables 2, 3).

The transitions shown in the RPs of the six test dynamics are evidently coincident with the shift points identified by the other methods. Here we used the CPA of each line in the RPs as a first approximation of a *Recurrence Quantification of Transitions* (RQT, sub-segmentation of the RPs). The TFD patterns shown in Figures 1(1–6G) demarcate transitions or interruptions of the frequency amplitudes which can be seen by the naked eye, but there is no immediate quantification available which could be applied to these patterns. The quantification which reduces the pattern to a single time series is given by the IF method.

Different methods applied to the data were able to identify change or transition points, but few of them are able to identify precursors or early warnings of critical transitions. One of the methods, DC, showed increased values just before the transition occurred [see Figures 1(3E,4E)], which corresponds to the theoretical concept of critical instabilities preparing phase transitions. There is empirical evidence that increased dynamic complexity precedes transitions and predicts good outcome (Schiepek et al., 2001, 2014; Haken and Schiepek, 2010; Olthof et al., 2019a). For short term predictions of suicidal crises DC may be used as an early warning signal for suicidal attempts or suicidal ideations (Fartacek et al., 2016). It should be noted that in the third empirical case [Figure 1(6A)], no increased DC could be found because the “transient relapse” transition was not characterized by preceding critical fluctuations.

### Implications for Clinical Practice and Research

Real-time monitoring of the therapeutic progress is getting increasingly popular and has been adopted by mental health providers all over the world (Schiepek et al., 2016c). Evidence is accumulating that the resulting time series are nonlinear and contain discontinuous jumps (see section “Introduction”). Importantly, it has been shown that such discontinuities have a clinical impact. For example, Helmich et al. (2020) report that sudden gains and nonlinear trajectories of the therapeutic progress were significantly more frequently observed in treatment responders. Likewise, increasing fluctuations have repeatedly been shown to improve treatment outcome as they indicate possibilities for the patient to reorganize (Schiepek et al., 2020). Identifying abrupt changes in an objective way is not trivial, especially in cases where the transition is not obvious to the eye. Combining several methods as proposed in this paper not only guarantees objectiveness but also gives important hints toward the validity of the change points. Moreover, the methods under investigation here allow not only to detect shifts of the mean and/or variance, but other properties like changes of the frequency, or of patterns in general. The study therefore considerably extends the common change point analyses. Once points of transitions have been identified in an objective and valid way, they can be used to look for precursors, i.e., variables that change before the system switches into another state. Successful identification of these indicators preceding change would, of course, be highly relevant for clinical practice. In any way, being aware of a change in the psychological system of a client can guide the practitioner through the therapeutic process as described by the Generic Principles (Haken and Schiepek, 2010).

### Limitations and Strengths

This study may be seen as a first step onto the development of an algorithm which could objectively identify phase transitions or phase transition-like phenomena (order transitions). We tested a limited number of methods applied to a limited number of data sets. Next steps have to include much more simulation runs and also much more empirical time series to test the converging results of the methods. A bigger number of cases would allow for a statistical testing of the results. A bigger number of simulation runs with simulated control parameter dynamics (comp. example 3 in our study) would produce some evident and clear-cut transitions, but also ambiguous dynamics and dynamics without transitions. It would be necessary to evaluate the discriminative validity of the methods for differentiating pronounced, less pronounced and non-existing transitions, and also for different degrees of signal-to-noise ratios.

Another limitation of our preliminary work concerns the use of surrogate data testing of the time series analysis (Theiler et al., 1992; Prichard and Theiler, 1994; Rapp et al., 1994; Schreiber and Schmitz, 2000; Schreiber and Schmitz, 2008). Random surrogates, which are created by random shuffling of the original series of the data points, destroy the nonlinear characteristics of a time series but preserve the linear ones, even the frequency distribution by creating phase-randomized surrogates. Based on a multitude of surrogates, a statistical test of the results from the original time series against the distribution of the results from the surrogates is possible. This was not done in our study because its focus was on the comparison of methods and their convergent validation, not in the statistical testing of the results using big numbers of cases and surrogate data distributions. Both will be realized in the next step of the validation project.

We included a very restricted number of methods which were known as appropriate for the analysis of short non-stationary real-world time series. Other methods exist (see the section “Perspectives”) and should be tested for their sensitivity to detect critical transitions.

The strength of the study is to combine proved methods for the identification of critical transitions in simulated and empirical data sets. This goes beyond what is called “eye balling” and allowed for a first evidence of the comparative robustness and validity of the methods.

### Perspectives

Most of the methods used in this convergent validity test seem to be promising and are used in routine process monitoring of psychotherapy. In the SNS, DC, PE, RP, and SPA are implemented (Schiepek et al., 2018). Beyond this, many other methods are available for the identification of critical transitions and related early warnings, which were published in different disciplines (e.g., climate research, ecology, brain dynamics, and physiology). In a current research project based on about 1.000 simulation runs and 1.000 empirical cases – each of which was documented by multiple time series assessed by daily TPQ-ratings – the methods will be tested for feasibility and for sensitivity to detect transitions.

Basically, all methods for the identification of chaotic dynamics can be used for the identification of transitions from regularity or noise to chaos or from one type of chaos to another. One is the titration of chaos with added white noise of increasing standard deviation, until its nonlinearity gets undetected by a particular indicator at a limiting value of the noise limit (Poon and Barahona, 2001). Others are the well-known Lyapunov exponents, or more specifically, the spectrum of Lyapunov exponents, one for each dimension of a m-dimensional system (Wolf et al., 1985; Rosenstein et al., 1993). More important than static measures of chaoticity are dynamic ones, which calculate the largest Lyapunov exponent of a time series within a gliding window (Kowalik et al., 2001) and by this identify changes of predictability (exponential divergences of nearby trajectories in an attractor) (Kowalik et al., 1997), or the Pointwise Correlation Dimension (Skinner, 1992; Skinner et al., 1994) which uses each vector point of an attractor as a reference for calculating the correlation dimension D2. By this, it allows for the identification of transitions in dynamic processes (Kowalik et al., 1997). Developments of methods for the estimation of fractal dimensionality (D2) and Lyapunov exponents for the identification of non-stationary systems are presented in Strunk and Schiepek (2006; for applications in psychotherapy research see Haken and Schiepek, 2010; Schiepek et al., 2016a).

Scheffer et al. (2009) and Dakos et al. (2012) report on a variety of indicators of next to transition dynamics, such as increased variance, critical slowing down with indicators like increased autocorrelations or lag1 autoregression coefficients, as well as extended skewness, kurtosis and conditional heteroscedasticity in the distribution of the time series values. Although the authors refer primarily to catastrophe theory, critical instability with increased sensitivity to perturbations and critical slowing down – the relaxation to stable attractors takes longer time after perturbations – are core concepts of far-from-equilibrium phase transitions as developed in Synergetics (Haken, 1977). Whereas critical instabilities can be identified by measures like DC, the detection of increased autocorrelation may require much longer time series (Bence, 1995) which usually are not available in psychotherapy research. Further indicators of critical slowing down are spectral reddening (higher variation at low frequencies) close to transitions, and increases in short- and mid-term memory measured by Detrended Fluctuation Analysis (Dakos et al., 2012).

An interesting characteristics of system dynamics approaching an attractor shift is flickering which is not only produced by internal or external perturbations or by sensitivity to noise, but by transient, short-term shifts between different alternative regimes before the system breaks its symmetry into one of these attractors (Schiepek et al., 2017). Methods proposed by Dakos et al. (2012) for the identification of dynamics driven by repeatedly crossing the domains of attraction of alternative states is threshold AR(*p*) models and potential analysis. Potential analysis is a technique for deriving the shape of the underlying potential of a system assuming that a time series may be approximated by a stochastic potential equation including a term for polynomial potentials of even order. The order of the best-fit polynomial in essence reflects the number of potential system states identified along the time series (Livina et al., 2011; Dakos et al., 2012). Finally, the emergence of scale-invariant power-law distributions (de Felice and Giuliani, 2020) is a precursor of transitions.

Chen et al. (2016) proposed an inconsistence index based on the computational method of Hidden Markov models. The inconsistence index measures the probability of a time point in a discrete time series being a switching point from a stationary Markov process to a time-varying Markov process. Healthy, before-transition states should be stationary Markov processes with high resilience and robustness to perturbations, whereas pre-transition states to diseases should be low resilient and more sensitive to perturbations (time-varying Markov processes). The inconsistence index passes a threshold during a pre-transition stage from a healthy pre-disease state to a disease state. Molenaar et al. (2009) used an extended Kalman Filter with iteration and smoothing to estimate time-varying parameters in non-stationary (non-ergodic) state-space models of empirical data.

One of the prominent methods for the identification of pattern transitions is RP. Unfortunately, the existing quantitative indicators of the features of a RP characterize the plot as a whole (Recurrence Quantification Analysis). They grasp important features as the percent of a plot filled with recurrent points (%Recurrent), percent of recurrent points forming diagonal lines (%Determinism), the Shannon information entropy of the line length distribution (Entropy), the length of the longest line segment (MaxLengths), and a measure of the paling of recurrent points away from the central diagonal (Trend) (Giuliani et al., 2001; Webber and Zbilut, 2005). Wallot et al. (2016) proposed sophisticated methods of Recurrence Quantification Analysis for multidimensional time series. However, up to our knowledge indicators of sub-segments and shifts of the dynamic qualities are not yet available. Calculating the change points of each line in the RPs may be a first approximation to *Recurrence Quantification of Transitions* (RQT), but other quantitative indicators of shifting recurrence patterns and sub-segmentation strategies should be developed, what is the topic of one of our currents projects.

There are promising methods which are not based on point-like measures but on spatio-temporal data sets. A method proposed by Goswami et al. (2018) transforms the sequential data of a spatial grid into a sequence of probability density functions which can be analyzed, e.g., by networks of recurrence probabilities. Ecosystems evidently undergo specific self-organized spatial patterns as they approach a critical transition, detectable by the analysis of coherences in the spatial structure (Scheffer et al., 2009). A method for the identification of changing coherence patterns is Spatiotemporal Stochastic Resonance (STSR) which measures the stability of spatial patterns displaying resonance-type dependency on noise amplitudes (Hütt et al., 2002).

The application of these methods onto psychotherapy and other psychological data sets needs for modifications, developments, and rigid testing. In a next step, they should be applied to big data sets created from computer simulations and empirical process monitoring with the aim of selecting the most valid ones on the way to a robust algorithm for detecting phase transitions and their precursors.

## Data Availability Statement

The datasets generated for this study are available on request to the corresponding author.

## Author Contributions

GS wrote the manuscript and supervised the process of data analysis. HS and KV realized the time series analysis. GF, SS, and MB provided the data of an empirical case and discussed the manuscript. CF discussed the manuscript and checked the writing. WA contributed to the organization of the research process and provided the data of an empirical case. All authors contributed to the article and approved the submitted version.

## Conflict of Interest

The authors declare that the research was conducted in the absence of any commercial or financial relationships that could be construed as a potential conflict of interest.

## Footnotes

1 A hypersphere is a high-dimensional sphere, i.e., a set of points at a constant distance from a given point called its center. Here, since we used three embedding dimensions, the hypersphere is equivalent to a sphere.

2 www.psysim.at
